# Voltage-gated sodium channels in diabetic sensory neuropathy: Function, modulation, and therapeutic potential

**DOI:** 10.3389/fncel.2022.994585

**Published:** 2022-11-17

**Authors:** Stephanie Bigsby, Joseph Neapetung, Verónica A. Campanucci

**Affiliations:** Department of Anatomy, Physiology and Pharmacology (APP), College of Medicine, University of Saskatchewan, Saskatoon, SK, Canada

**Keywords:** diabetic neuropathy (painful), Na_*v*_ channels, modulation, therapeutics, sensory neurons

## Abstract

Voltage-gated sodium channels (Na_*V*_) are the main contributors to action potential generation and essential players in establishing neuronal excitability. Na_*V*_ channels have been widely studied in pain pathologies, including those that develop during diabetes. Diabetic sensory neuropathy (DSN) is one of the most common complications of the disease. DSN is the result of sensory nerve damage by the hyperglycemic state, resulting in a number of debilitating symptoms that have a significant negative impact in the quality of life of diabetic patients. Among those symptoms are tingling and numbness of hands and feet, as well as exacerbated pain responses to noxious and non-noxious stimuli. DSN is also a major contributor to the development of diabetic foot, which may lead to lower limb amputations in long-term diabetic patients. Unfortunately, current treatments fail to reverse or successfully manage DSN. In the current review we provide an updated report on Na_*V*_ channels including structure/function and contribution to DSN. Furthermore, we summarize current research on the therapeutic potential of targeting Na_*V*_ channels in pain pathologies, including DSN.

## Introduction

Diabetic neuropathies are amongst the most common chronic complications of diabetes, affecting approximately 50–60% of diabetic patients (Boulton et al., 2005; Zakin et al., 2019). Neuropathy results from diabetes-induced damage to peripheral nerves, and up to 30% of those patients develop a sensory form of neuropathy (diabetic sensory neuropathy or DSN) (Boulton et al., 2005; Tesfaye et al., 2013). DSN can present with a wide spectrum of clinical symptoms from tingling, numbness, weakness, and sensory loss, to exacerbated pain perception (Callaghan et al., 2012; Kobayashi and Zochodne, 2018). Patients suffering from DSN can display one or more types of stimulus-evoked pain, such as exacerbated responses to noxious (hyperalgesia) or innocuous (allodynia) stimuli (Hong et al., 2004; Tesfaye et al., 2010). DSN involves sensory abnormalities that most commonly manifest in a symmetrical “stocking-and-glove” distribution, being experienced in the distal extremities (e.g., hands and feet) and progresses proximally towards the torso over the course of diabetes (Callaghan et al., 2012). Consequently, DSN patients are at a higher risk of developing “diabetic foot,” a condition characterized by the development of unhealing foot ulcers, which can eventually contribute to lower limb amputations (Callaghan et al., 2012; Kobayashi and Zochodne, 2018). Ultimately, pathological damage to the peripheral nerves in DSN is debilitating, resulting in a considerable reduction in patient quality of life. Despite a strong relationship between blood glucose levels and neuropathy, the underlying mechanisms contributing to the pathology of DSN remain unclear. Unfortunately, no therapeutic strategy has succeeded in halting, reversing or preventing the development of DSN (Dyck et al., 1999; Callaghan et al., 2012; Kobayashi and Zochodne, 2018).

Some symptoms characteristic of DSN, such as numbness and hyposensitivity, are usually associated with long-standing diabetes and the progressive degeneration of peripheral nerves and loss peripheral innervation (Chantelau, 2015). Although painful symptoms are also consequence of nerve damage and inflammation, they have been linked to biochemical changes affecting membrane proteins and signaling pathways in sensory neurons. Amongst the many proteins that may contribute to the development and/or progression of DSN, voltage-gated sodium channels (Na_*V*_) have been particularly considered. Na_*V*_ channels are essential for the initiation and propagation of action potentials, and thus, are responsible for electrical signaling (Bagal et al., 2015). Activation of Na_*V*_ channels carry the increase in sodium conductance during action potentials, allowing the movement of sodium ions down their electrochemical gradient and into the cell following local depolarization (Catterall, 1992; De Lera Ruiz and Kraus, 2015). This large influx of sodium ions causes further depolarization, which activates more Na_*V*_ channels triggering a positive feedback leading ultimately to the rising phase of the action potential (Chong and Ruben, 2008). Various Na_*V*_ channels participate in pain perception, which has made them subjects of extensive investigation into the pathophysiology of pain, including their contribution to pain symptoms in DSN (Dib-Hajj et al., 2010).

## Na_*V*_ function is intimately linked to channel structure and tissue distribution

Na_*V*_ channels are heteromeric complexes composed of a pore-forming α subunit and at least one associated β subunit (Bagal et al., 2015; O’Malley and Isom, 2015). The α subunit family members (Na_*V*_1.1 - 1.9) are highly homologous in amino acid sequence and display tissue specificity throughout the body (De Lera Ruiz and Kraus, 2015). They are encoded by the genes SCN1A-SCN5A and SCN8A-SCN11A (Hoeijmakers et al., 2015). These subunits are large single polypeptide chains of approximately 260 kDa, composed of 4 homologous domains each containing a voltage sensor, a pore region, and a selectivity filter (De Lera Ruiz and Kraus, 2015).

Na_*V*_ channels are commonly characterized by their differential sensitivity to tetrodotoxin (TTX), Na_*V*_1.1–1.4, 1.6, and 1.7 are inhibited by this toxin with an IC_50_ in the low nanomolar concentration range, and thus are considered TTX-sensitive (TTX-S) (Baer et al., 1976; De Lera Ruiz and Kraus, 2015). TTX-S channels carry faster activating and inactivating currents (Wang et al., 2011; Yin et al., 2016). Meanwhile, Na_*V*_ 1.5, 1.8, and 1.9 are inhibited by TTX with an IC_50_ in the micromolar range, and hence are considered as TTX-resistant (TTX-R) (Baer et al., 1976; De Lera Ruiz and Kraus, 2015). TTX-R currents show slower activation and inactivation kinetics (Wang et al., 2011; Yin et al., 2016). Na_*V*_1.1 and 1.3 are expressed in both the central (CNS) and the peripheral (PNS) nervous systems (Cummins and Rush, 2007; Bagal et al., 2015). Na_*V*_ 1.1 expression in peripheral dorsal root ganglion (DRG) neurons is high in large-diameter neurons, moderate in medium-diameter neurons, and low in small diameter neurons. A small portion of Na_*V*_1.1-positive neurons co-express isolectin B4 (IB4), a marker for nociceptive neurons, suggesting this isoform may play some role in pain transmission (Wang et al., 2011). Na_*V*_1.3 expression in DRGs is highest in the embryonic period of development, and downregulates postnatally (Cummins and Rush, 2007; Bennett et al., 2019). Na_*V*_1.2, on the other hand, is highly expressed in the CNS. It is predominantly found in dendrites, unmyelinated axons, and pre-myelinated axons (Wang et al., 2011) predominantly in the embryonic period (Wang et al., 2011; Bennett et al., 2019). Other Na_*V*_ subunits are almost exclusively expressed in embryonic muscle (Bennett et al., 2019), Na_*V*_ 1.4 in skeletal (Cummins and Rush, 2007) and Nav 1.5 in the cardiac muscle (Rogers et al., 2006; Cummins and Rush, 2007).

The rest of the Na_*V*_ isoforms (Nav 1.6–1.9) are expressed within the DRGs and play important roles in nociception (Rush et al., 2007; Chen et al., 2018). Na_*V*_1.6 expression in DRG, as well as in motor neurons, is preferentially targeted to the nodes of Ranvier in myelinated fibers and along unmyelinated C- fiber (Wittmack et al., 2005; Bennett et al., 2019). Nav1.7 is the most expressed TTX-S isoform, mostly found in small diameter Aδ and C-fibers (Black et al., 1996; Toledo-Aral et al., 1997; Berta et al., 2008; Dib-Hajj et al., 2010, 2013; Ho and O’Leary, 2011), including 85% of functionally identified nociceptors (Djouhri et al., 2003). Nav1.8 and Nav1.9, are also highly expressed in nociceptive neurons (Akopian et al., 1997; Dib-Hajj et al., 1998; Bennett et al., 2019). Na_*V*_1.8 is the major contributor to the rising phase of the action potential; carrying 80–90% of the inward sodium current during the upstroke of the action potential (Renganathan et al., 2001), and its ability to rapidly recover from inactivation allows for repetitive, high frequency firing (Waxman, 2012). Na_*V*_1.9 is almost exclusively expressed in the PNS (Cummins and Rush, 2007), particularly in C-fiber nociceptors and with moderated expression in medium diameter (Aδ-fibers) and low in large diameter (Aβ-fibers) (Rogers et al., 2006; Priest and Kaczorowski, 2007). Na_*V*_ 1.9 contribute to the amplification of subthreshold inputs, but do not contribute to the upstroke or amplitude of the action potential (Bennett et al., 2019). These are considered threshold channels due to their activation at hyperpolarized potentials (near the resting membrane potential), where other Na_*V*_ channels remain inactive (De Lera Ruiz and Kraus, 2015; Bennett et al., 2019).

Most Na_*V*_ channels contain associated β subunits; although they were traditionally considered as “auxiliary,” growing evidence suggests they are critical for channel function. The β subunits promote channel trafficking (Schmidt and Catterall, 1986; Chen et al., 2002), and modulate channel biophysical properties (Calhoun and Isom, 2014). The 4 known β subunits (β1- β4) (Qin et al., 2003; Brackenbury and Isom, 2011) are encoded by genes SCN1B-SCN4B. Mutations in these genes, as well as changes in expression levels, have been linked to the development of many isoform-specific pathologies such as epilepsy, Huntington’s disease, cardiac arrhythmias, and various neuropathies (Meadows et al., 2002; Lucas et al., 2005; Oyama et al., 2006; Medeiros-Domingo et al., 2007; Brackenbury and Isom, 2011; Alsaloum et al., 2019). These subunits have a much smaller molecular weights than their α counterparts (30–40 kDa, Priest and Kaczorowski, 2007), and are members of the immunoglobulin superfamily of cell adhesion molecules (De Lera Ruiz and Kraus, 2015). β1 and β3 noncovalently bind to the α-subunit, while β2 and β4 covalently attach to the α-subunit via disulfide bonds (Namadurai et al., 2015). The immunoglobulin domain modulates expression and gating properties of α subunits, while the transmembrane domain influences their voltage dependence (Rogers et al., 2006; De Lera Ruiz and Kraus, 2015). β subunits are found in the CNS, PNS, heart, and skeletal muscle (excepting β2) (Isom et al., 1992; Brackenbury and Isom, 2011). β1 and β4 can modulate channel kinetics by enhancing the rate of inactivation and recovery from inactivation (Namadurai et al., 2015). β4 is the promoter of the resurgent current, which flows through channels that reopen in response to negative voltage changes due to the decay of the macroscopic sodium current, but when the inactivation gates have yet to close (Raman and Bean, 1997). In contrast, β3 is mostly expressed in heart muscle (Brackenbury and Isom, 2011) and has been linked to cardiac arrhythmias and cardiac conduction problems (O’Malley and Isom, 2015).

## Contribution of Na_*V*_ channels to diabetic sensory neuropathy

The induction of experimental diabetes in rodents using streptozotocin (STZ), a pancreatic β-cell-specific cytotoxin, results in hyperglycemia due to lack of insulin production (Islam and Loots, 2009). STZ-induced diabetic animals develop painful neuropathy accompanied by a typical increase in Na_*V*_1.3, 1.6, and 1.7 expression (Cummins et al., 1998, 2001; Herzog et al., 2003; Hong et al., 2004) and DRG neuron hyperexcitability (Hirade et al., 1999). The pivotal role of Na_*V*_1.7 in nociception (Black et al., 1996; Toledo-Aral et al., 1997; Berta et al., 2008; Ho and O’Leary, 2011; Dib-Hajj et al., 2013) has been well documented, particularly by the effects of mutations. While gain-of-function mutations in the Na_*V*_1.7 subunit lead to hereditary pain disorders, such as primary erythromelalgia, paroxysmal extreme pain disorder and small fiber neuralgia (De Lera Ruiz and Kraus, 2015; Tibbs et al., 2016), loss-of-function mutations lead to congenital insensitivity to pain (De Lera Ruiz and Kraus, 2015). In the DRG of diabetic rodents, Nav1.7 channel expression increased robustly and triggered evoked pain symptoms of thermal hyperalgesia and mechanical allodynia (Hong et al., 2004). Consistently, symptoms of thermal hyperalgesia and mechanical allodynia in these animals were attenuated by the miRNA-mediated knockdown of the Na_*v*_1.7 α subunit (Chattopadhyay et al., 2012). Furthermore, electrophysiological studies in DRG neurons from diabetic rats revealed that the TTX-S current showed an increased current density, a negatively shifted voltage-dependent activation, and delayed inactivation kinetics (Hong and Wiley, 2006). The latter are consistent with expression and function changes reported in Na_*V*_1.7, which is the predominant TTX-S isoform expressed in DRG neurons (Black et al., 1996; Toledo-Aral et al., 1997; Berta et al., 2008; Ho and O’Leary, 2011; Dib-Hajj et al., 2013).

Isoforms Na_*V*_1.2, 1.3, and 1.9 are upregulated in the DRG of STZ-induced diabetic rats (Hong and Wiley, 2006). The detection of Na_*V*_1.2 and 1.3 in adult diabetic rodents contrasts with physiological expression levels of these subunits, since they are normally higher in embryonic neurons (Cummins and Rush, 2007; Wang et al., 2011; Bennett et al., 2019). The latter suggest that the diabetic environment triggers a pathological resurgence in the expression of these embryonic channels. It has been reported that Na_*V*_1.3 is upregulated in the adult spinal cord of rats after peripheral nerve injury (Black et al., 2004; Cummins and Rush, 2007) and in the DRG after axotomy (Hains et al., 2003). And more importantly, the knockdown of Na_*V*_1.3 expression successfully reduced evoked tactile allodynia and hyperexcitability in the dorsal horn neurons of STZ-induced diabetic rats (Tan et al., 2015). These findings suggest that Na_*V*_1.3 could be a suitable therapeutic target in the treatment of DSN.

Contrasting with other Na_*V*_s, the expression levels of Na_*V*_1.6 and 1.8 decreased in DRG homogenates from diabetic rats (Hong et al., 2004). Although the mechanisms for this downregulation is still unclear, it has been reported that reactive oxygen species (ROS) reduced Na_*V*_1.8 peak current in DRG neurons (Schink et al., 2016). This finding is consistent with the well documented generation of ROS in diabetes from mitochondria as well as by enzymatic and non-enzymatic glucose oxidation, and constitute the most explored hypothesis for the effects of diabetes on the nervous system (Russell et al., 2002; Vincent et al., 2005; Tomlinson and Gardiner, 2008; Campanucci et al., 2010; Chandna et al., 2015; Lam et al., 2018; Momeni et al., 2021).

Much less, however, is known about the possible contribution of β subunits to DSN. Genetic analysis of the β2 subunit gene from a diabetic patient, who presented symptoms of painful neuropathy, revealed a gain-of-function mutation of an aspartic acid substituted by asparagine mutation, D109N. This point mutation lead to DRG neuron hyperexcitability (Alsaloum et al., 2019). These findings were in line with the increase in β2 subunit expression in neuropathic pain models of injured and uninjured DRG neurons (Pertin et al., 2005), highlighting this subunit as a potential player in the development of varied pain pathologies.

## The therapeutic potential of Na_*V*_ channels

Table 1 summarizes the current knowledge on approved therapeutic strategies for DSN targetting Na_*V*_ channels, as well as mechanisms with the potential to modulate Na_*V*_ function in DSN. Tricyclic antidepressants (TCAs), such as amitriptyline, imipramine, nortriptyline, and duloxetine, are Food and Drug Administration (FDA) approved drugs that are effective for the treatment of painful forms of DSN (Berger et al., 2005). A recent report (Horishita et al., 2017) demonstrate that the analgesic effects of some of these drugs in neuropathic pain pathology is mediated by the inhibition of Na_*V*_ channels. Particularly, they strongly inhibited Na_*V*_1.7, and Na_*V*_1.8 in DRG, as well as upregulated Na_*V*_1.3 in the DRG of models of peripheral nerve injury (Lindia et al., 2005; Fukuoka et al., 2008). These drugs also inhibited, but in a weaker fashion, Na_*V*_ channels highly expressed in the CNS, such as Na_*V*_1.6 and Na_*V*_1.2 (Horishita et al., 2017). Another therapeutic approach, not first considered as targeting Na_*V*_ channels, is the pungent ingredient in “hot” chili peppers, capsaicin. Capsaicin is an agonist of the transient receptor potential vanilloid 1 (TRPV1), a non-selective cation channel (Caterina et al., 1997). TRPV1 plays a central role in pain transduction, and its inhibition alleviates thermal hyperalgesia and mechanical allodynia in animal models of peripheral nerve injury (Walker et al., 2003; Kasama et al., 2007; Sugimoto et al., 2013). Interestingly, capsaicin can also induce analgesia. In fact, the topical application of capsaicin has been used for the treatment of localized pain (Deal et al., 1991; Hersh et al., 1994; Gratton and Cusson, 1995; Winocur et al., 2000; Melis et al., 2019), and the use of capsaicin as a therapeutic approach for diabetic patients with symptoms of painful neuropathy has been recently reviewed by Dludla et al. (2022). It is well accepted that repeated exposure to capsaicin induces a calcium-mediated desensitization of TRPV1 channels (Jancsó et al., 1967; Hains et al., 2003). However, the analgesic effects of capsaicin are mediated in part by the inhibition of Na_*V*_ channels through second messengers, such as cAMP (Liu et al., 2001). Although, the identity of the specific Na_*V*_ isoforms modulated by capsaicin remain to be explored. Another approach targetting Na_*V*_ channels in DSN is the use of the antithrombotic agent cilostazol (Cheng et al., 2022). The interest on cilostazol for DSN symptoms stems from its known protection of human endothelial cells *via* activation of ERK1/2 and p38 MAPKs (MAPK) (Lim et al., 2009), and neuroprotection in animal models of cerebral ischemia (Iwama et al., 2007). When tested on diabetic rats, oral administration of cilostazol successfully decreased withdrawal threshold to mechanical stimuli and attenuated neuropathic pain symptoms. More importantly, it reduced expression levels of multiple Na_*V*_ channels (Na_*V*_1.1, 1.2, 1.6, and 1.7); and restored expression levels of Na_*V*_1.8, which was markedly reduced in STZ rats. The anticonvulsant gabapentin, has also been testes to treat DSN. Gabapentin induced analgesia in STZ-induced diabetic rats, it successfully reverted mechanical allodynia and thermal hyperalgesia in these animals, which correlated with reduced expression of the Na_*V*_1.7 isoform and phosphorylated ERK1/2 in DRG neurons (Zhang et al., 2013). Similar findings come from pioglitazone, a proliferator-activated receptors (PPARs) agonist usually prescribed to type 2 diabetic patients, and ranolazine, an adjuvant in chronic angina medication (Elkholy et al., 2020). Both drugs successfully reverted symptoms of mechanical allodynia and thermal hyperalgesia in type 2 diabetic rats. Furthermore, these drugs individually reduced the expression levels of Na_*V*_1.7 in DRG neurons to control levels (Elkholy et al., 2020).

**TABLE 1:** Modulation of Na_*V*_ expression and channel function as treatment for DSN.

	Effect	DSN	Findings	Citation	DOI or PMID
**FDA drugs**					
TCAs	Analgesia	Y	Stronger inhibition of DRG Nav1.3, Nav1.7, and Nav1.8 in DRG, and weaker inhibition of Nav1.2 and Nav1.2 in *Xenopus oocytes.*	Horishita et al., 2017	*10.1007/s00210-017-1424-x*.
Capsaicin	Analgesia	Y	cAMP-mediated inhibition of Nav channel isoforms	Liu et al., 2001	*10.1152/jn.2001.85.2.745*
Cilostazol	Analgesia	Y	In diabetic rats it decreased evoked pain symptoms. Downregulation of Nav1.1, 1.2, 1.6 and 1.7; and restored expression levels of Nav1.8, which was downregulated in STZ rats.	Cheng et al., 2022	*10.3389/FPHAR.2021.771271*
Gabapentin	Analgesia	Y	In STZ rats, it reverted evoked pain symptoms and downregulated Nav1.7 and p-ERK1/2 in DRG neurons.	Zhang et al., 2013	*10.1016/J.BRAINRES.2012.11.032*
Ranolazine and Pioglitazone	Analgesia	Y	Reversion of evoked pain symptoms in T2D rats. Reduction of sciatic TNF-α and 1L-1b, and downregulation of Nav1.7 channels. Upregulation of PPAR-γ in spinal cord.	Elkholy et al., 2020	*10.1016/J.LFS.2020.117557*
**Kinases**					
p38 MAPK	Increase	N^&^	Enhancement of TTX-R currents in DRG, and increased Nav1.7 conductance.	Jin and Gereau, 2006; Black et al., 2008; Nemoto et al., 2010	*10.1523/JNEUROSCI.3858-05.2006*; *10.1002/ANA.21527*; *10.1016/J.EJPHAR.2010.04.048*
p38, ERK1/2, JNK MAPKs	Increase	N^&^	TNF-α mediated increase of TTX-R currents, mostly Nav1.8 in DRG neurons from a model of femoral artery occlusion.	Li et al., 2020	*10.1152/AJPREGU.00338.2019*
TNF-α and p-NFκB	Increase	Y	Nav1.7 in the DRG on STZ-induced diabetes	Huang et al., 2014	*10.1016/J.NEUINT.2014.05.012*
p38 MAPK and PKC	Reduction	Y	δ-opioid receptor activation led to reduced phosphorylation of p38 MAPK and PKC and prevented Nav1.7 upregulation.	Chattopadhyay et al., 2008	*10.1523/JNEUROSCI.5530-07.2008*
**Antibody**					
SVmab1	Reduction	N	Nav1.7 current inhibition in mouse and human DRG.	Lee et al., 2014; Bang et al., 2018	*10.1016/J.CELL.2014.03.064*; *10.1007/S12264-018-0203-0*
**ECS**					
AJA	Reduction	N	Inhibited Nav1.2 - 1.5, Nav1.7 - 1.8, and β4 subunit-mediated resurgent currents in Nav1.5 channels.	Foadi et al., 2014	*10.1213/ANE.0000000000000188*
AEA	Reduction	N^&^	Inhibition of Nav 1.2, 1.6 - 1.8. Inhibition of β4 subunit-mediated resurgent currents in Nav1.7	Theile and Cummins, 2011; Okura et al., 2014	*10.1124/mol.111.072751*; *10.1213/ANE.0000000000000070*
AEA, AM 404 and WIN 55,212-2,	Reduction	N	Direct inhibition of TTX-S currents.	Nicholson et al., 2003	*10.1016/S0006-8993(03)02808-7*
THC	Reduction	N	Reduction of Nav currents and conductance in the nodes of Ranvier in frogs.	Strichartz et al., 1978	310454
CBD	Reduction	N	Inhibition of Nav1.1-1.7 in HEK-293 cells and iPSC neurons. Inhibition of Nav1.4 in diaphragm (muscle). Inhibition of Nav1.7 and Nav1.8 in DRG neurons. The formation of the Nav-CBD complex in Alphaproteobacteria inhibited Nav functions.	Sula et al., 2017; Ghovanloo et al., 2019; Ghovanloo et al., 2021; Zhang and Bean, 2021; Ghovanloo et al., 2022	*10.1038/NCOMMS14205*; *10.1074/jbc.RA118.004929*; *10.1085/jgp.202012701*; *10.1523/JNEUROSCI.3216-20.2021*; *10.1111/bph.15833*
CBD	Cell protection	N	Restoration of Nav1.5 gating defect, which causes cytotoxicity in epithelial cells.	Fouda et al., 2020	*10.1111/bph.15020*
					

The table includes information about FDA approve drugs currently use in the treatment of DSN, signaling kinases, antibody therapy, and cannabinoids. DSN, diabetic sensory neuropathy; T2D, type two diabetes; Y, yes, tested in diabetic patients or animal models of DSN; N, not tested in DSN; N^&^, not tested in DSN but in models of neuropathic pain.

Targeting signaling kinases such as those from the mitogen-activated protein kinase (MAP) and protein kinase (PK) families in the context of DSN, is supported by reports highlighting their role regulating Na_*V*_ expression and function in other pain pathologies. For instance, in human painful neuromas, multiple Na_*V*_ isoforms (Na_*V*_1.1 - 1.3, Na_*V*_1.6 - 1.9) co-localized with the activated MAPKs p38 and extracellular signal-regulated kinases 1 and 2 (ERK1/2), suggesting these signaling proteins could potentially modulate Na_*V*_ channel function in painful neuropathies (Black et al., 2008). In fact, in chromaffin cells, p38 and ERK1/2 primed Na_*V*_1.7 function and increased ion conductance (Nemoto et al., 2010). Activation of the p38 MAPK pathway by tumor necrosis factor-α (TNF-α) enhanced TTX-R currents in isolated DRG neurons and induced mechanical hypersensitivity in mice, which was prevented by the pharmacological inhibition of p38 (Jin and Gereau, 2006). Similarly, in DRG neurons from a model of femoral artery occlusion, TNF-α mediated the increase of TTX-R currents, mostly through Na_*V*_1.8; and this effect was prevented by the pharmacological inhibition of p38, ERK1/2, and c-Jun N-terminal kinases (JNKs) (Li et al., 2020).

Consistently, in STZ-induced diabetic rats with symptoms of DSN, the upregulation of Na_*V*_1.7 in the DRGs was prevented by inhibiting the synthesis or by blocking the action of TNF-α and p-nucleus factor-kappa B (p-NFκB)(Huang et al., 2014). In DRG neurons exposed to hyperglycemic conditions *in vitro*, the enkephalin-mediated activation of δ-opioid receptors prevented Na_*V*_1.7 upregulation. The later was accompanied by reduction of phosphorylation of p38 MAPK and PKC (Chattopadhyay et al., 2008).

A provocative new approach proposes the use of monoclonal antibodies to inhibit Na_*V*_1.7 channels. This novel strategy revealed that the antibody SVmab1, selectively inhibited Na_*V*_1.7 currents in mouse and human DRG neurons (Lee et al., 2014; Bang et al., 2018). Moreover, the antibody effectively supressed inflammatory and neuropathic pain in mouse models, and unveiled a significant role of Na_*V*_1.7 in itch sensation (Lee et al., 2014), suggesting this strategy may be effective in the management of chronic itch in humans (Bang et al., 2018). Although, this antibody therapy has yet to be tested in the context of DSN, the ability of SVmab1 to reduce excitatory transmission in pain-sensitive neurons may relief diabetes-related pain symptoms.

More recently, targeting Na_*V*_ channels through the endocannabinoid system (ECS) has been proposed as a therapeutic tool in pain pathologies. The ECS is composed of endocannabinoids, their receptors, and the enzymatic pathways required for endocannabinoids’ synthesis. The endocannabinoids, N arachidonoylethanolamine (AEA), and 2-arachidonoylglycerol (2-AG) bind to two well-characterized G-protein coupled receptors cannabinoid receptors 1 and 2 (CB1) and 2 (CB2) (Howlett et al., 2004). Numerous animal and clinical studies have shown the potential of synthetic and naturally occurring cannabinoids to effectively attenuate inflammatory and neuropathic pain, including DSN pain (Howlett et al., 2004; Rahn and Hohmann, 2009). Some of the beneficial effect of cannabinoids on DSN could be explained by their action on Na_*V*_ channel function. The synthetic cannabinoid ajulemic acid (AJA), which has been reported to induce analgesia in inflammatory pain in humans (Burstein et al., 2004), inhibited Na_*V*_1.2 - 1.5, Na_*V*_1.7 - 1.8 channels, as well the β4 subunit-mediated resurgent currents in Na_*V*_1.5 channels (Foadi et al., 2014). Similarly, the endocannabinoid anandamide (AEA) has been reported to inhibit β4 subunit-mediated resurgent currents in Na_*V*_1.7 (Theile and Cummins, 2011). Cannabinoids are known to modulate presynaptic Ca^2+^ and K^+^ channels (Nicholson et al., 2003), but also to inhibit Na_*V*_ channels either through CB_1_ receptor signaling or by their direct action on the channel protein. AEA, as well as the synthetic cannabinoids AM 404 and WIN 55,212-2, directly bound to TTX-S Na_*V*_ channels (Nicholson et al., 2003). Moreover, AEA was reported to inhibit Na_*V*_ 1.2, 1.6 - 1.8 (Okura et al., 2014) by unknown mechanisms. The phytocannabinoid Δ9-tetrahydrocannabinol (THC), the principal psychoactive constituent of cannabis, has previously been reported to decrease peak Na^+^ current and conductance in the nodes of Ranvier in frogs (Strichartz et al., 1978). Furthermore, cannabidiol (CBD), a phytocannabinoid lacking psychoactive effect, has been reported to block Na_*V*_ channels. CBD inhibited human Na_*V*_1.1-1.7 currents expressed in human embryonic kidney 293 (HEK-293) cells and in induced pluripotent stem cell (iPSC)-derived neurons. The mechanisms of action of CBD seemed to be indirect, mediated by its interaction with membrane lipids resulting in loss of Na_*V*_ channel activity (Ghovanloo et al., 2019). Consistently, in DRG neurons, CBD inhibited Na_*V*_1.7 leading to reduced neuronal excitability (Ghovanloo et al., 2022), a mechanisms with relevant potential in pain pathologies. CBD also inhibited Na_*V*_1.4 and 1.8 by similar mechanisms. In muscle, CBD stabilized the inactivated state of Na_*V*_1.4 (Ghovanloo et al., 2021). In the DRGs, CBD showed preferential binding to the slow inactivated state of Na_*V*_1.8, which directly inhibited repetitive firing of nociceptors (Zhang and Bean, 2021). Moreover, CBD was shown to block Na_*V*_ currents by physically interacting with the channels. The high-resolution crystal structure of the Na_*V*_-CBD complex was studied in Na_*V*_ channels from *M. marinus* (Sula et al., 2017), which exhibits similar function, sequence and structural homologies to mammalian Na_*V*_ channels (Sula and Wallace, 2017; Sula et al., 2017), demonstrated that CBD interacts with the channel at a novel site at the location of the central hydrophobic cavity of the channel (Sait et al., 2020). Furthermore, CBD has been shown to restore gating defects in Na_*V*_ 1.5 caused by reactive oxygen species in high glucose conditions, which protected against high glucose-induced oxidative stress and cytotoxicity in the Chinese hamster ovary (CHO) epithelial cell line (Fouda et al., 2020). More importantly, the beneficial effect of engaging the ECS has been documented in patients with painful DSN. A randomized, double-blinded, placebo-controlled crossover study revealed improved spontaneous and evoked pain scores between placebo and patients receiving treatment (Wallace et al., 2015), which may be mediated at least in part by the modulation of Na_*V*_ channels.

## Conclusion

**FIGURE 1:**
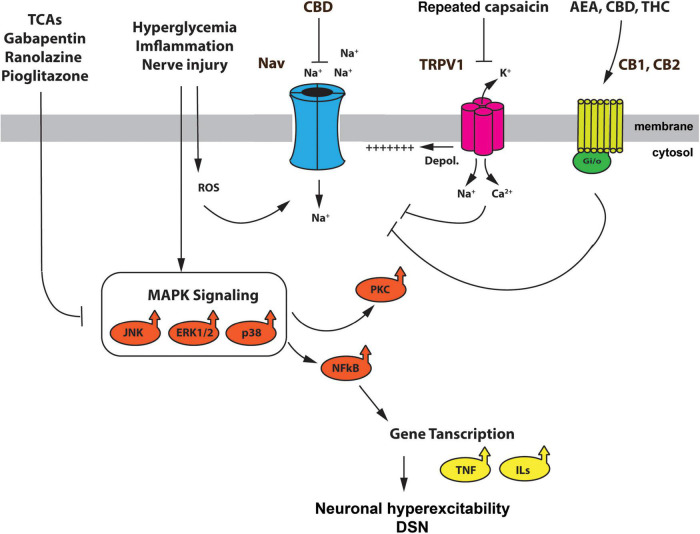
Schematic representation summarizing the mechanisms modulating Na_*V*_ channel function in DSN, and the effect of therapeutic agents. Hyperglycemia, nerve injury, and inflammation activate the MAPK kinase pathway and generate reactive oxygen species (ROS). The p38, ERK1/2 and JNK MAPKs, as well as ROS, have been reported to mediate upregulation and enhancement of different Na_*V*_ channel subunits. Furthermore, MAPK signaling leads to PKC activation and expression NFkB. The latter supports the expression of interleukins and TNF-a further enhancing an inflammatory environment in DSN. TRPV1 channels, which are upregulated by hyperglycemia, inflammatory mediators, and ROS (Lam et al., 2018; Nair et al., 2021), depolarize DRG neurons and contribute to excitability in DSN (Jin et al., 2004; Hong and Wiley, 2005; Melli and Höke, 2009) by assisting the cell in reaching threshold voltage for activation of Na_*V*_ channels. The use of FDA approved drugs for the treatment of DSN such as TCA, gabapentin, ranolazine and pioglitazone, reduce the activation of the MAPK pathway, preventing the upregulation of some Na_*V*_ channels, such as Na_*V*_1.7. Furthermore, tonic (repeated) application of capsaicin prevents the upregulation of Na_*V*_ channels by cAMP mediated signaling pathways. Agonist of the ECS, such as AEA, THC, and CBD, can prevent Na_*V*_ channel upregulation by actions through they receptors (CB1, CB2), or by direct action on the Na_*V*_ channel. Although these effects of cannabinoids have yet to be tested in the context of DSN, they do, however, provide a mechanistic explanation of some of the beneficial effects of cannabinoid therapy in diabetic patients with symptoms of DSN. TCAs, tricyclic antidepressants; ROS, reactive oxygen species; ECS, endocannabinoid system; AEA, N arachidonoylethanolamine, CBD, cannabidiol; THC, Δ9-tetrahydrocannabinol; CB1, cannabinoid receptor 1; CB2, cannabinoid receptor 2.

In the current review, we focused on Na_*V*_ channels and their role in DSN, not only due to their unequivocal relevance in cell excitability, but also because several recent studies point at Na_*V*_s as potential therapeutic targets in pain pathologies. Here we discussed the important role that some Na_*V*_s play in DSN and their yet largely potential for pain management in diabetes. Novel strategies using Na_*V*_s as a therapeutic tool may involve (1) FDA approved drugs with effects on Na_*V*_ channel function; (2) signaling kinases that regulate Na_*V*_ expression/function; (3) monoclonal antibody therapy; and (4) modulation by the ECS. Figure 1 summarizes the therapeutic potential of Na_*V*_ channels for the treatment of DSN symptoms, including strategies that have yet to be tested in the context of diabetes.

## Author contributions

SB and VC conceptualized the manuscript. SB, JN, and VC wrote the manuscript. JN and VC edited the manuscript. All authors contributed to the article and approved the submitted version.
